# Tissue Plasminogen Activator in Trabecular Meshwork Attenuates Steroid Induced Outflow Resistance in Mice

**DOI:** 10.1371/journal.pone.0072447

**Published:** 2013-08-19

**Authors:** Sandeep Kumar, Shaily Shah, Hai Michael Tang, Matthew Smith, Teresa Borrás, John Danias

**Affiliations:** 1 Department of Cell Biology, SUNY Downstate Medical Center, Brooklyn, New York, United States of America; 2 Department of Ophthalmology, SUNY Downstate Medical Center and the SUNY Eye Institute, Brooklyn, New York, United States of America; 3 Mount Sinai School of Medicine, New York, New York, United States of America; 4 Department of Ophthalmology, University of North Carolina, Chapel Hill, North Carolina, United States of America; University of South Alabama Mitchell Cancer Institute, United States of America

## Abstract

Tissue plasminogen activator, a serine protease encoded by the PLAT gene is present in the trabecular meshwork (TM) and other ocular tissues and has been reported to be downregulated by treatment with steroids in vitro. Steroids are known to cause changes in outflow facility of aqueous humor in many species. In the present study, we tested whether overexpression of PLAT can prevent and/or reverse the outflow facility of mouse eyes treated with steroids. Animals received bilateral injection with 20 µl of triamcinolone acetonide (TA) (40mg/ml) suspension subconjunctivally to induce outflow facility changes. Some animals received unilateral intracameral injection with 2 µl of adenoviral suspension [3-4x10^12^ virus genomes per milliliter (vg/ml)] carrying sheep PLAT cDNA (AdPLAT) either concurrently with TA injection or one week after TA injection, whereas others received bilateral intracameral injection with 2µl of adenoviral suspension (9x10^12^ vg/ml) carrying no transgene (AdNull) concurrently with TA injection. Animals were sacrificed one week after AdPLAT or AdNull treatment. Endogenous mRNA expression levels of mouse PAI-1 and MMP-2, -9 and -13 were also measured using qRT-PCR. Outflow facility one week after AdPLAT administration was increased by 60% and 63% respectively for animals that had not or had been pretreated with steroids. Overexpression of PLAT significantly upregulated expression of PAI-1, MMP-2, -9 and -13 compared to the levels found in TA only treated eyes. These findings suggest that overexpression of PLAT in TM of mouse eyes can both prevent and reverse the decrease in outflow facility caused by steroid treatment and is associated with upregulation of MMPs.

## Introduction

Tissue plasminogen activator (tPA) is a serine protease, localized in the uveal microvasculature, corneal endothelium, corneal epithelium and the trabecular meshwork (TM) of the eye [1]. tPA enzymatic activity has also been measured in aqueous humor (AH) in dog, calf, monkey [2] and human eyes [3]. It catalyzes the conversion of plasminogen (an inactive proenzyme of the fibrinolytic system), to plasmin, a protease involved in the degradation of a variety of proteins. Plasmin is a critical component of the fibrinolytic pathway but also activates several pro matrix-metalloproteinases (pro-MMPs) to their active form [4]. MMPs are a family of zinc containing proteases which play an important role in the degradation of extracellular matrix (ECM) proteins. It has been suggested that these enzymes are necessary to maintain normal (AH) outflow. Decrease in these enzymes could result in the accumulation of ECM components in the TM leading to alteration in aqueous outflow [5,6]. The effect of tPA-mediated MMP activation is inhibited either at the level of tPA by specific inhibitors [plasminogen activator inhibitor-1 (PAI-1) and plasminogen activator inhibitor-2 (PAI-2)] or at the level of plasmin mainly by alpha 2-antiplasmin [7]. Proper balance between tPA and its inhibitors is critical for homeostatic maintenance.

Glucocorticoids (GC) are well known to cause an increase in intraocular pressure (IOP) which is a major risk factor for glaucoma [8,9]. Approximately 30-40% of patients treated with GC topically or systemically develop ocular hypertension. The severity of disease depends on the GC potency, pharmacokinetics, duration of treatment and route of administration and steroid responders have a higher predisposition for developing glaucoma than nonresponders. Moreover, nearly 90% of patients with preexisting primary open-angle glaucoma (POAG) respond to steroid treatment with further elevation of IOP [10–12].

Although the exact mechanism by which steroid induces elevation in IOP is not well understood, chronic steroid treatment results in increased aqueous humor outflow resistance and is associated with morphological and biochemical changes in the TM [13–15]. These changes include inhibition of expression and activity of MMPs and downregulation of PLAT, the gene encoding for tPA [16–18]. The inhibition of those molecules can be what causes decrease in the breakdown of the ECM of the TM resulting in increased outflow resistance.

To test this hypothesis, we overexpressed PLAT in the TM, utilizing a recently introduced mouse model of steroid induced outflow facility change [19]. We also analyzed by qPCR the levels of PAI-1 and MMPs 2, 9 and 13 to determine whether any effects are caused via MMPs.

## Materials and Methods

### Animals

Three-month old female mice of C57BL/6 strain were used in this study. The animals were kept under a 12-hour light/12-hour dark cycle and fed ad libitum. This study was carried out in strict accordance with the ARVO Statement for the Use of Animals in Ophthalmic and Vision Research and was approved by the Institutional Animal Care and Use Committee (IACUC). The protocol was approved by the Committee on the Ethics of Animal Experiments of the State University of New York (SUNY) Downstate Medical Center, Brooklyn, NY.

### Adenoviral vector construction

**Figure 1:**
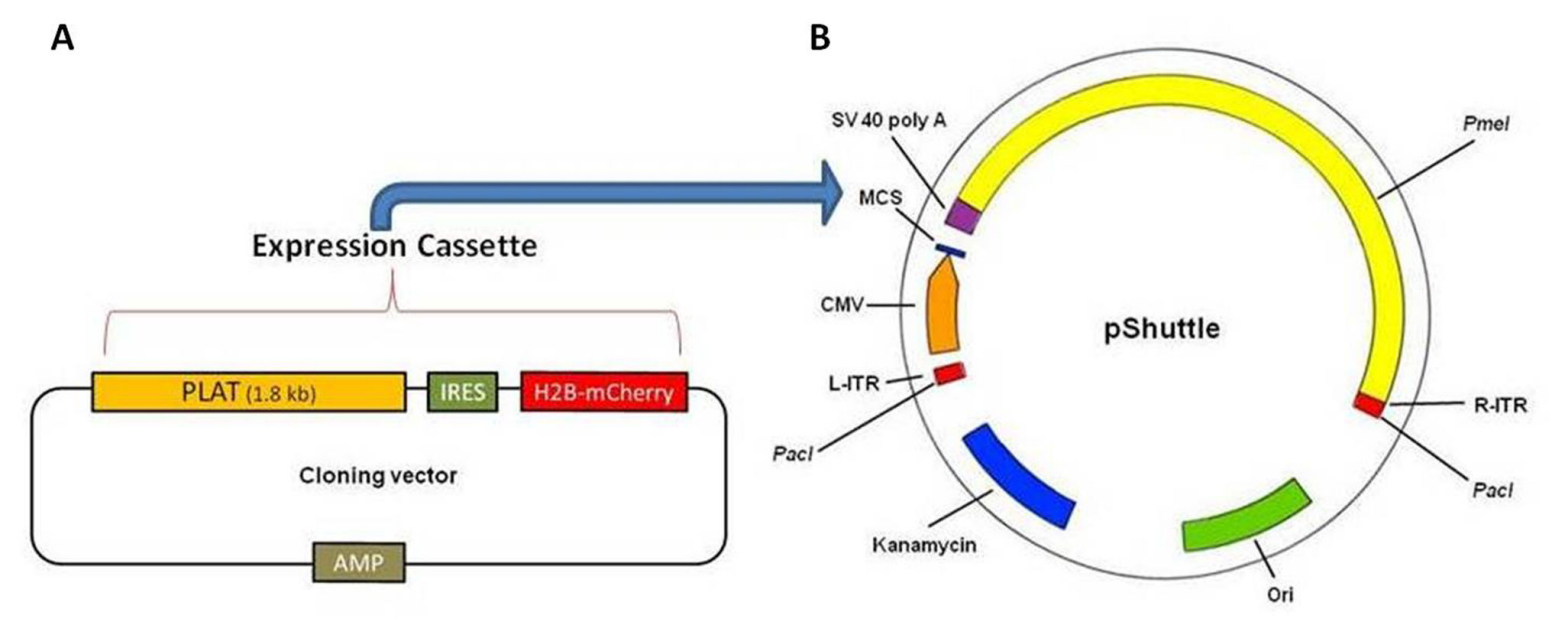
Map of AdPLAT Construct. PLAT gene was amplified from cDNA synthesized from RNA extracted from sheep brain tissue. The expression cassette (**A**) was constructed by placing amplified and gel purified PLAT cDNA adjacent to a human histone B (H2B) tagged fluorescent reporter gene (mCherry) with internal ribosome entry site (IRES) in a suitable cloning vector. The expression cassette was then subcloned downstream of a CMV promoter at the multiple cloning site of the shuttle vector (**B**) and used to generate adenovirus (AdPLAT).

The full coding region of the sheep PLAT mRNA (1.8 kb) (NCBI reference sequence number *XM_004021803.1*) was amplified using cDNA synthesized from RNA extracted from sheep brain tissue. Amplification was performed using Phusion High-Fidelity PCR Master Mix with HF Buffer (NEB Inc, Ipswich, MA, USA). Primer sequences used for amplification were as follows; forward primer: *5’-CTGAAGACAACGCCTCTGGAGGA-3’* and reverse primer: *5’-TGGGAGAAGCGGGGTCTCTGTG-3’*. PCR product was subjected to sequence analysis to confirm the sheep PLAT mRNA sequences. The expression cassette was constructed by placing amplified and gel purified PLAT cDNA adjacent to a human histone B (H2B) tagged fluorescent reporter gene (mCherry) with internal ribosome entry site (IRES) in a suitable cloning vector (Figure 1A). The expression cassette was then subcloned downstream of a CMV promoter at the multiple cloning site of the shuttle vector [20] (Figure 1B). All cloning reactions were performed using In-Fusion HD cloning kit (Clontech, Mountain View, CA, USA) according to the manufacturer’s protocol.

### Adenovirus generation and titration

Adenovirus vectors carrying transgene (AdPLAT) and no transgene (AdNull) were generated by homologous recombination using the AdEasy Adenoviral Vector System (Stratagene, La Jolla, CA) [21]. Briefly, shuttle vectors containing transgene or no transgene were linearized with PmeI and electroporated into BJ5183-Ad1 cells for the recombination with the adenovirus backbone plasmid (pAdEasy1) according to manufacturer’s directions. The resultant vectors were amplified in *E. coli* competent cells XL10-gold (Stratagene, La Jolla, CA), purified, linearized with PacI and transfected into early-passage QBI-HEK 293A cells (Qbiogene, Montreal, Canada) for the production of the recombinants. High-titer viral stocks were obtained by propagation in the same cells and purification by double banding CsCl density centrifugation [21]. The collected viral band was desalted with NAP-5 columns (GE Healthcare, Piscataway, NJ) equilibrated with virus vehicle (0.01 M Tris pH 7.4, 1 mM MgCl_2_, 10% glycerol), aliquoted and stored at -80^o^C.

Physical particles of adenovirus were titrated as virus genomes per milliliter (vg/ml) by real-time PCR after extraction of the viral DNA using the DNeasy tissue kit (Qiagen, Valencia, CA). Viral infectivity was measured as infectious units/ml (IFU/ml) with a rapid titer kit (AdenoX; Clontech, Mountain View, CA) containing an antibody specific to the adenovirus hexon capsid protein produced only in infected cells [21].

### Steroid and adenoviral treatment

**Figure 2:**
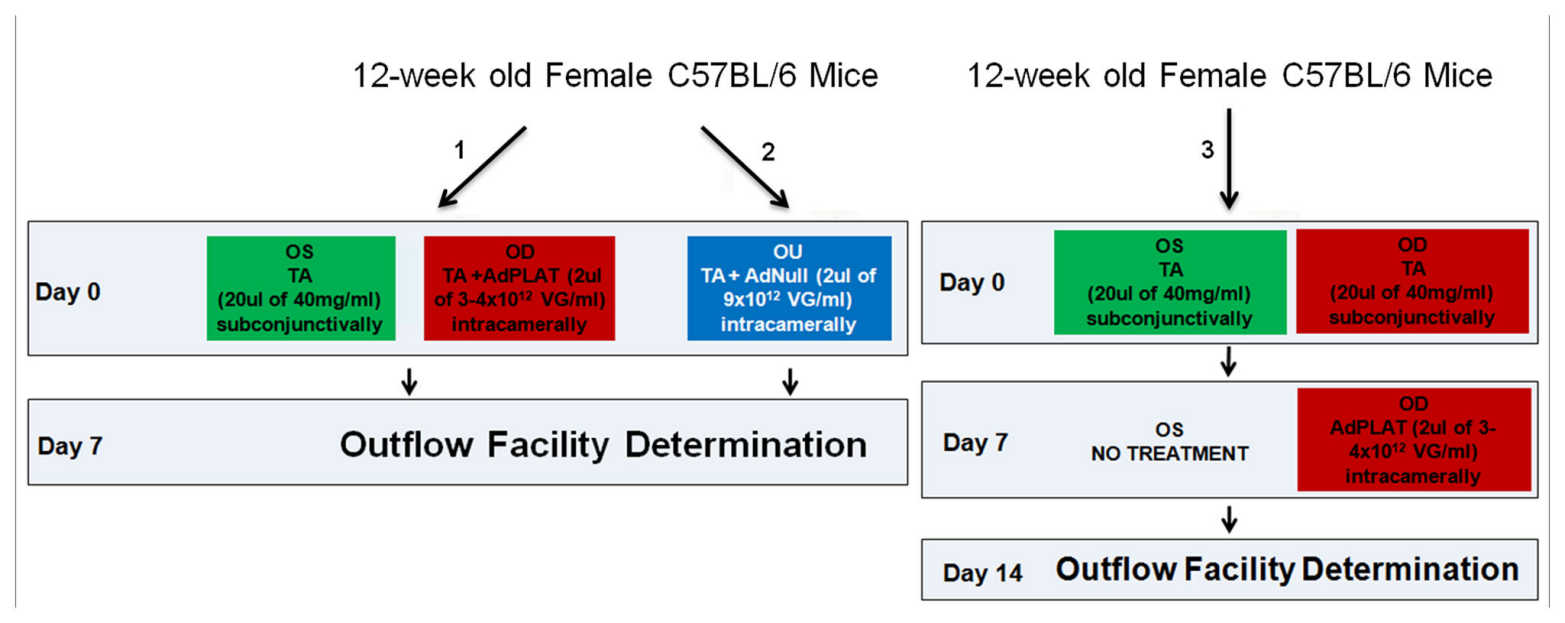
Experimental Design. 12 week-old C57BL/6 female mice received bilateral injections with 20 ul of triamcinolone acetonide (TA) suspension subconjunctivally. Animals were then divided into three treatment groups: 1) animals that also received unilateral intracameral injection with 2 ul of AdPLAT concurrently with TA injection while the contralateral eye remained uninjected, 2) animals that received bilateral intracameral injection with 2 ul of AdNull concurrently with TA injection, and 3) animals that received unilateral intracameral injection with 2 ul of AdPLAT one week after TA injection while the contralateral eye remained uninjected. Animals were sacrificed for outflow facility determination one week after AdPLAT or AdNull treatment.

All animals received bilateral injection with 20 µl of triamcinolone acetonide (TA) (40mg/ml) suspension subconjunctivally using a Hamilton syringe with a 26-gauge needle. Animals were then divided into three groups (Figure 2): 1) animals that received unilateral intracameral injection with 2 µl of AdPLAT (3-4x10^12^ vg/ml) concurrently with the TA injection while the contralateral eye remained uninjected, 2) animals that received bilateral intracameral injection with 2 µl of AdNull (9 x10^12^ vg/ml) concurrently with TA treatment, and 3) animals that received unilateral intracameral injection with 2 µl of AdPLAT one week after TA injection while the contralateral eye remained uninjected. The intracameral injections were performed using a Hamilton syringe with a 36-gauge stainless steel needle (WPI Inc, Sarasota, FL, USA). Animals were sacrificed one week after AdPLAT or AdNull treatment. All injections were performed under intraperitoneal anesthesia with ketamine/xylazine mix and topical anesthesia with 0.5% proparacaine. An additional group of naïve untreated mice was used for RNA extraction and served as naïve controls to determine ΔΔCt by qRT-PCR (ΔΔCt is defined in subsection ‘quantitative real-time PCR’, see below).

### IOP measurement

IOP was measured in mice preterminally with a rebound tonometer [22]. The animals were held in a custom-made restraint that does not compress the chest or neck while IOP is measured [23]. IOP measurements were performed after application of 0.5% proparacaine topical anesthesia. Five measurements were obtained per eye and averaged. IOP measurements were performed between 10 AM and 12 PM, to minimize the effect of diurnal IOP variation.

### Outflow facility determination

The animals were sacrificed by cervical dislocation under deep surgical anesthesia. The eyes were immediately enucleated and used for the determination of outflow facility. Outflow facility was determined using a constant flow method. Eyes were initially perfused at a flow rate of 0.1 µl/min and the system was allowed to run for 30-40 minutes to achieve stable baseline pressure. Flow rate was then increased sequentially to 0.13, 0.15, 0.2, 0.25, 0.3 µl/min. Pressures were recorded either continuously using a chart recorder or intermittently (every 5 minutes) using a digital recorder. The pressure readings at steady state at each flow rate were plotted and the slope of the regression line was used to calculate outflow facility as described previously [19].

### Visualization of mCherry expression under fluorescent microscope

After perfusion, mCherry expression in the TM was determined in all AdPLAT-injected eyes. The eyes were dissected on ice to isolate a rim of tissue containing the TM by removing most of the cornea, iris, and ciliary body. Flat mounts of the rims containing TM were observed in an epifluorescent microscope equipped with the appropriate filter sets to visualize mCherry expression. After observation, dissected rims were immediately immersed in RNA stabilizing agent (RNAlater, Ambion; Invitrogen, Carlsbad, CA).

### Tissue collection and Isolation of RNA

All perfused eyes were immersed in RNA stabilizing agent at 4°C overnight and stored at -20°C until RNA extraction. The eyes were dissected on ice in the presence of RNA stabilizing agent to isolate a rim of tissue containing the TM as described above. Rims containing TM from eyes of the same analysis group were pooled together (four at a time) to achieve sufficient amount of RNA for analysis. The rims were homogenized and total RNA was extracted using TRIzol reagent (Gibco/Invitrogen, Carlsbad, CA). Briefly, the tissue was homogenized in TRIzol and chloroform was added to separate proteins from RNA. After centrifugation, the RNA-containing supernatant was aspirated. The RNA was precipitated with isopropanol, washed with 75% ethanol, and resuspended in nuclease-free water. RNA concentrations were determined with a spectrophotometer (Nanodrop, Thermo Scientific, Wilmington, DE) and the 260/280-nm absorbance ratio calculated to determine RNA purity.

### Quantitative real-time PCR

The RNA samples were reverse transcribed with random hexamers to cDNA using a reverse transcription kit (Quantitect; Quiagen, Valencia, CA), in accordance with the manufacturer’s instructions. Quantitative real-time PCR (qRT-PCR) was performed using a commercial kit (SYBR Green RT-PCR Reagents Kit; Applied Biosystems, Carlsbad, CA). qRT-PCR was carried out in a ABI PRISM 7900HT sequence detector (Applied Biosystems, Carlsbad, CA). The mRNA expression of sheep PLAT transgene (allowing us to measure PLAT transgene transcription), and mouse endogenous matrix metalloproteinase-2 (MMP-2), matrix metalloproteinase-9 (MMP-9), matrix metalloproteinase-13 (MMP-13) and plasminogen activator inhibitor 1 (PAI-1) in the TM were investigated in eyes from all experimental groups. The mouse specific primer sequences used are listed in Table 1. Relative quantification of gene expression was performed using the standard curve method. Relative fold change was calculated by the formula 2^-ΔΔCt^ where Ct is defined as the cycle at threshold. ΔCt is defined as the difference in mean of Ct values derived from the target gene and the 18S gene (an internal control) and ΔΔCt is defined as ΔCt of the normalized assayed genes in the treated samples minus ΔCt of normalized assayed genes in the naïve control samples. Fold changes were compared to 1 by single group t-test.

**Table 1:** Primer sequences of genes analyzed by qRT-PCR.

**Gene**	**Primer**	**Sequence (5’- 3’)**	**Size (bp)**
PLAT	forward	*CAGTGCCCAGAAGGGTTCAT*	249
	reverse	*GTAGCACCAGGGCTTTGAGT*	
MMP-2	forward	*GACCATGCGGAAGCCAAGAT*	122
	reverse	*CCAGGTCAGGTGTGTAACCA*	
MMP-9	forward	*GTCCAGACCAAGGGTACAGC*	195
	reverse	*GCCTTGGGTCAGGCTTAGAG*	
MMP-13	forward	*AGTGCCTGATGTGGGTGAAT*	161
	reverse	*GTGGTGTCACATCAGACCAGA*	
PAI-1	forward	*GCTGTAGACGAGCTGACACG*	218
	reverse	*TAGGGCAGTTCCACAACGTC*	
18S	forward	*AGTCCCTGCCCTTTGTACACA*	69
	reverse	*GATCCGAGGGCCTCACTAAAC*	

### Statistical analysis

Statistical analysis was performed using GraphPad Prism (3.03) software. The differences in means were analyzed using one way analysis of variance (ANOVA) followed by Tukey’s multiple comparison test. Values of p<0.05 were considered to be statistically significant.

## Results

### PLAT expression in AdPLAT injected eyes

**Figure 3:**
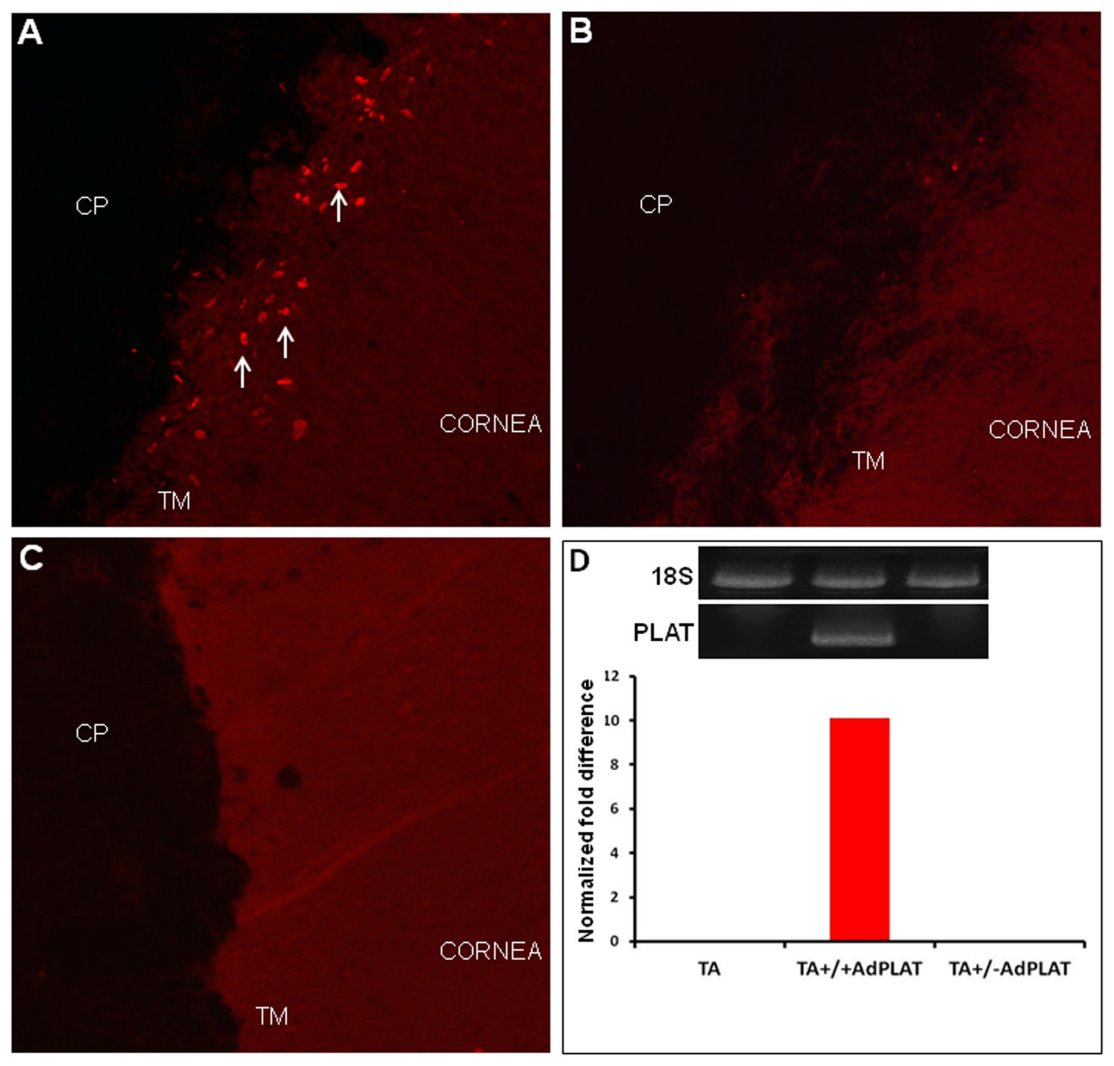
mCherry expression in flat mounts of TM in AdPLAT-injected Eyes. Flatmount of anterior segments of mouse eyes from (**A**) animal injected with AdPLAT that showed robust mCherry expression (TA+/+ AdPLAT) (**B**) animal with minimal mCherry expression despite injection with AdPLAT (TA+/-AdPLAT), and (**C**) treatment-naïve mouse. (**D**) qRT-PCR quantification of transgenic PLAT expression in mouse eyes. Levels of PLAT are undetectable in all eyes except those showing robust mCherry expression (TA+/+ AdPLAT). CP = ciliary processes, TM = trabecular meshwork. Arrows indicate mCherry-positive cells.

To verify PLAT expression, mCherry expression was visualized on flat-mounted TM immediately after outflow facility determination. mCherry expression was limited to the TM and distributed uniformly along the entire length of the TM. No mCherry expression was seen in the cornea or other tissues within the anterior segment (Figure 3A). Some AdPLAT injected eyes however, showed minimal (just one or two mCherry positive cells per eye) or no mCherry expression in the TM (Figure 3B). These eyes were analyzed as a separate group (TA+/-AdPLAT) from eyes which showed robust mCherry expression (TA+/+ AdPLAT). No mCherry expression was observed in naïve control eyes (Figure 3C). To further confirm PLAT expression and to determine whether expression also occurs in eyes without significant visible mCherry expression, qPCR was performed on RNA from TM tissue. The expression of sheep PLAT gene was observed in the TM of eyes receiving AdPLAT with robust mCherry expression but not in similarly treated eyes with minimal or no mCherry expression. TA alone injected eyes (TA) also showed no ovine PLAT gene expression by qPCR (Figure 3D).

### Effect of AdPLAT on IOP of TA injected eyes

In animals that received AdPLAT concurrently or one week after TA injection, no significant difference was observed in IOP among eyes injected with TA and AdPLAT showing robust mCherry expression, eyes injected with TA and AdPLAT showing minimal or no mCherry expression, and contralateral eyes injected with TA alone. IOP was also not significantly different between eyes injected with TA and AdNull (TA+AdNull) and eyes from all other groups. All eyes appeared normal on slit lamp examination.

### PLAT prevents and reverses decrease in outflow facility caused by TA

**Figure 4:**
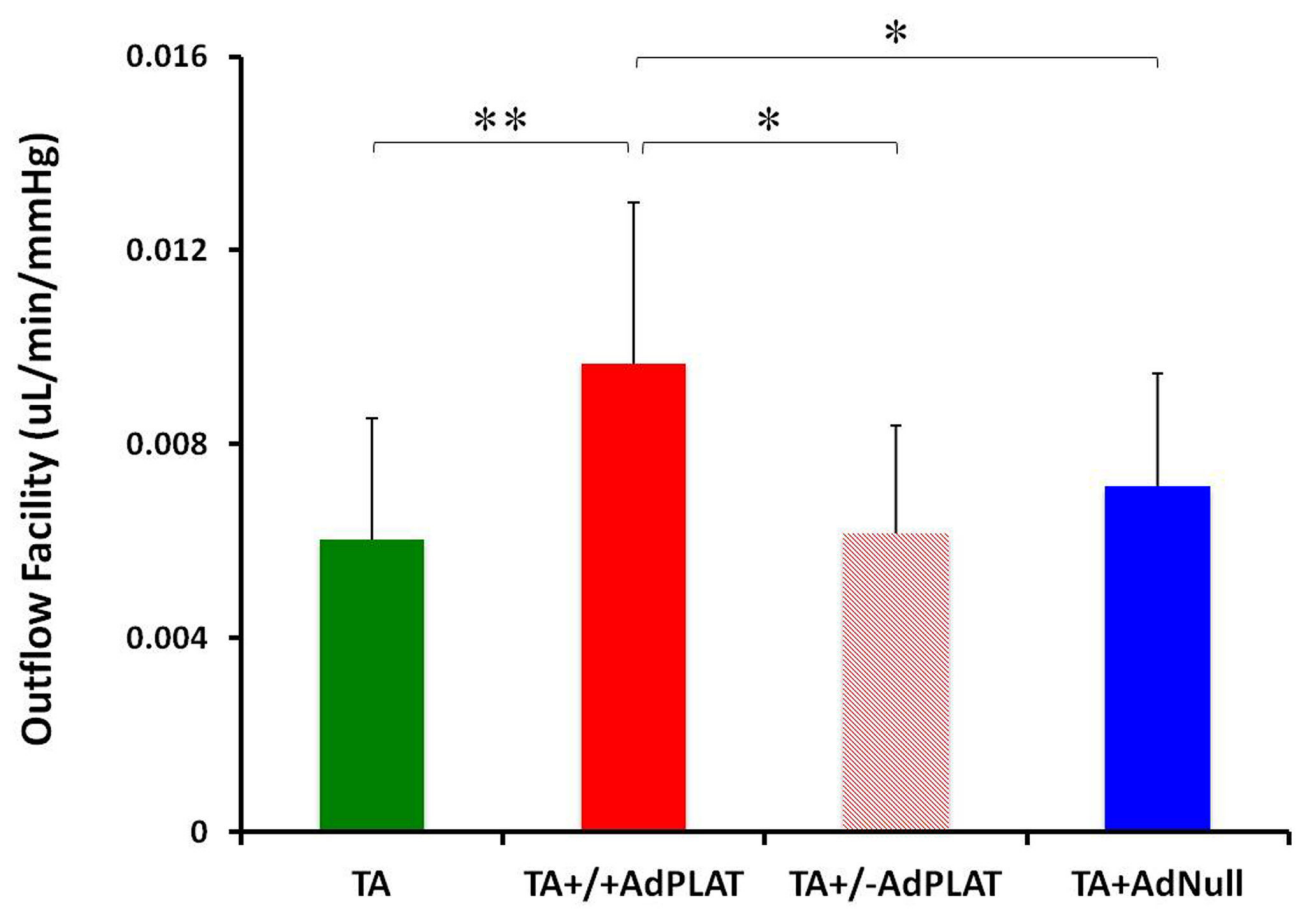
Outflow facility in TA and AdPLAT concurrently treated mouse eyes. Mean ± SD outflow facility in eyes receiving TA alone (TA, n=22), eyes injected with AdPLAT showing robust mCherry expression (TA+/+ AdPLAT, n=12), eyes injected with AdPLAT showing minimal or no mCherry expression (TA+/-AdPLAT, n=8), and eyes injected with AdNull (TA+AdNull, n=30). Group means are significantly different (ANOVA, p<0.01). Asterisks indicate significant differences on post hoc analysis, *p<0.05, **p<0.01.

In animals injected with AdPLAT concurrently with TA, the mean ± standard deviation (SD) outflow facility in AdPLAT injected eyes showing robust mCherry expression (TA+/+ AdPLAT) (n=12), AdPLAT injected eyes showing minimal or no mCherry expression (TA+/-AdPLAT) (n=8), and contralateral eyes injected with TA alone (TA) (n=22) was 0.0097 ± 0.0033, 0.0062 ± 0.0022, and 0.0060 ± 0.0025, respectively, while eyes that received both TA and AdNull concurrently (TA+AdNull) (n=30) had outflow facility of 0.0071 ± 0.0023. TA+/+AdPLAT eyes had a 60%, 56%, and 35% increase in outflow facility compared to TA, TA+/-AdPLAT, and TA+AdNull eyes, respectively. One-way ANOVA revealed significant difference (p<0.01) in outflow facility among TA+/+ AdPLAT, TA+/-AdPLAT, TA and TA+AdNull eyes. Post-hoc analysis showed a significant increase in outflow facility of TA+/+ AdPLAT eyes compared with TA+/-AdPLAT eyes (p<0.05), TA eyes (p<0.01), and eyes receiving both TA and AdNull (p<0.05). No significant difference in outflow facility was detected between TA+AdNull eyes, TA eyes or TA+/-AdPLAT eyes (Figure 4).

**Figure 5:**
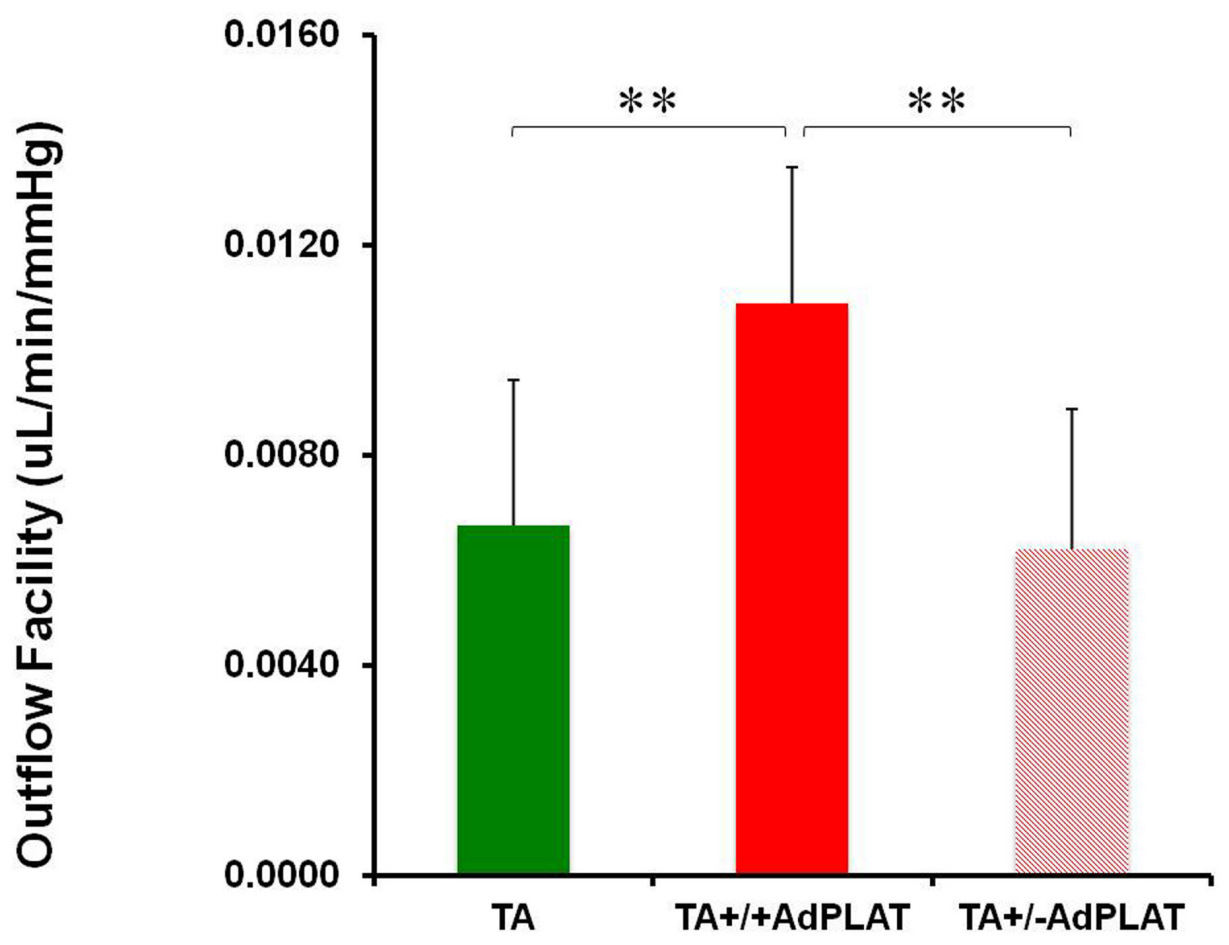
Outflow facility of eyes receiving AdPLAT one week after TA injection. Mean ± SD outflow facility in eyes receiving TA alone (TA, n=24), eyes injected with AdPLAT showing robust mCherry expression (TA+/+ AdPLAT, n=6), and eyes injected with AdPLAT showing minimal or no mCherry expression (TA+/-AdPLAT, n=11). Group means are significantly different (ANOVA, p<0.01). Asterisks indicate significant differences on post hoc analysis, **p<0.01.

In animals receiving AdPLAT one week after TA injection, the mean ± SD outflow facility in TA+/+ AdPLAT (n=6), TA+/-AdPLAT (n=11) and TA (n=24) eyes was 0.0109 ± 0.0026, 0.0062 ± 0.0027, and 0.0067 ± 0.0028 respectively. TA+/+AdPLAT eyes in this treatment arm had a 75% and 63% increase in outflow facility compared to TA+/-AdPLAT eyes and TA eyes respectively. Outflow facility was significantly different in TA+/+ AdPLAT eyes, TA+/-AdPLAT eyes and TA eyes (ANOVA, p<0.01). Post hoc analysis revealed a significant increase (p<0.01) in outflow facility of TA+/+ AdPLAT eyes compared with TA+/-AdPLAT and TA eyes. There was no significant difference in outflow facility among eyes receiving TA alone and TA+/-AdPLAT eyes (Figure 5).

### PLAT induces expression of PAI-1, MMP-2, MMP-9 and MMP-13

**Figure 6:**
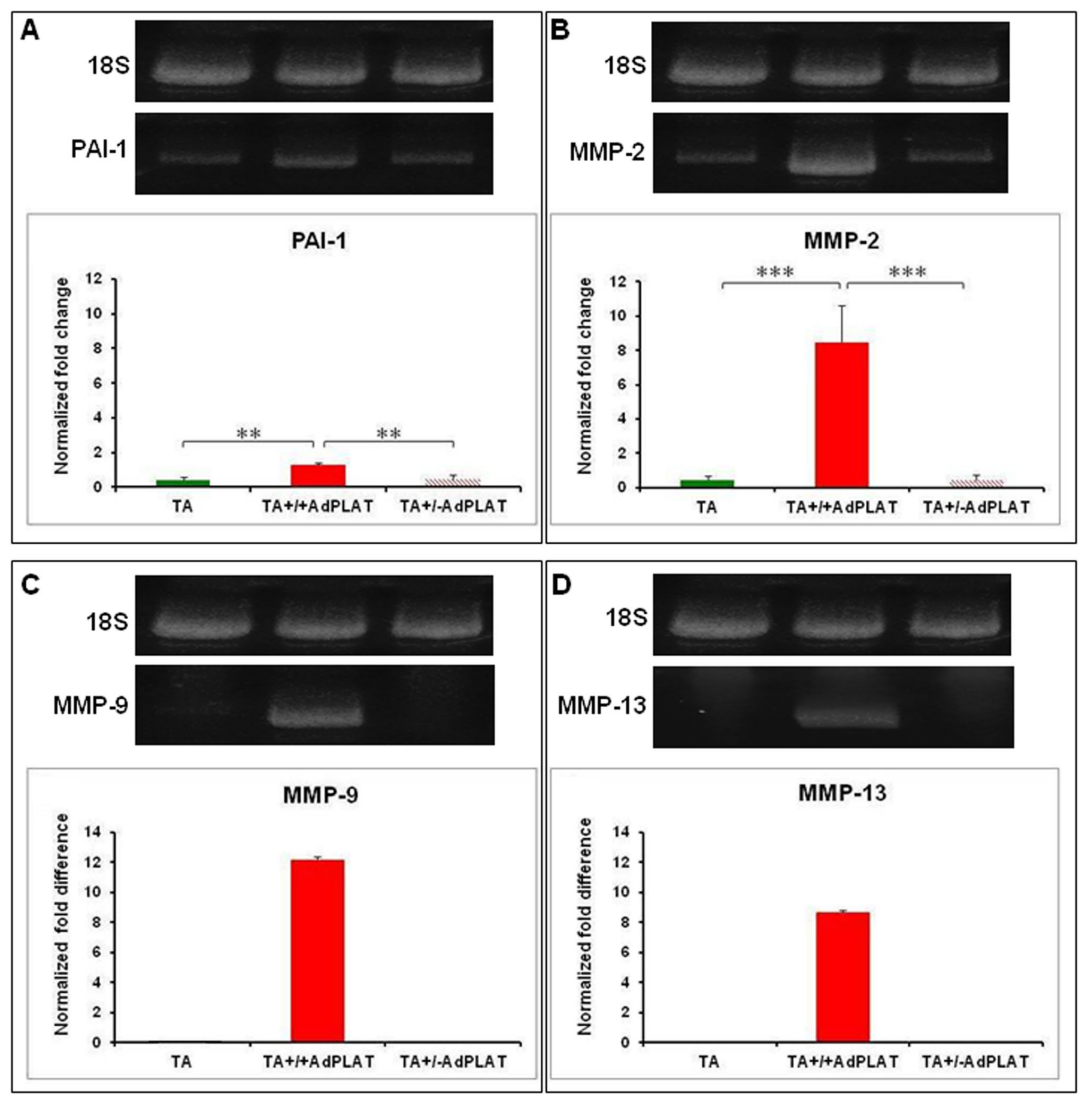
Gene expression changes. Normalized fold change (mean ± SD) of (**A**) PAI-1 and (**B**) MMP-2 expression and normalized fold difference of (**C**) MMP-9 and (**D**) MMP-13 expression in TA eyes (n=10), TA+/+ AdPLAT eyes (n=9), and TA+/-AdPLAT eyes (n=10) from animals receiving AdPLAT one week after TA injection. Group means are significantly different (ANOVA PAI-1, p<0.01, MMP-2, p<0.001). Asterisks indicate differences on post hoc analysis, **p<0.01, ***p<0.001. No mRNA was detected for MMP-9 and MMP-13 in the TA or TA+/-AdPLAT groups.

PAI-1and MMP-2 expression was significantly different (ANOVA, p<0.01 and p<0.001 respectively) among TA+/+ AdPLAT, TA+/-AdPLAT and TA treated eyes that had been treated with TA one week prior to AdPLAT administration. Post hoc analysis revealed that TA+/+ AdPLAT eyes had significantly higher (p<0.01) relative PAI-1 expression compared to TA+/-AdPLAT eyes and TA eyes (Figure 6A). The expression of MMP-2 was also significantly higher (p<0.001) in the TM of TA+/+ AdPLAT eyes compared to eyes from TA+/-AdPLAT and TA groups (Figure 6B). Compared to TA-alone injected eyes, the expression of PAI-1 was increased by 3.1 ± 1.1-fold and expression of MMP-2 was increased by 20.2 ± 11.6-fold in TA+/+ AdPLAT eyes.

MMP-9 and MMP-13 expression was also upregulated in TA+/+ AdPLAT eyes. However because their expression levels were beyond the detection limit in the naïve control group as well as in all other groups, the data could not be subjected to statistical analysis (Figure 6 C and D).

## Discussion

Glucocorticoid-induced IOP elevation has been an increasingly big problem with the increased use of intraocular steroids. Patients with POAG receiving various formulations of GC therapy have been shown to be particularly prone to developing further elevation in IOP [9,10]. The mechanism by which GCs cause increase in IOP is through increased outflow resistance in the TM, which is associated with alteration in ECM turnover [24,25]. ECM turnover is in part maintained by the regulation of proteolytic enzymes of the fibrinolytic pathway and MMP family [5,6]. GCs have been shown to alter these pathways in the TM and other ocular tissues by inhibiting the expression and activity of MMP and PLAT [16–18]. We previously have shown that PLAT is downregulated by ~40% in the TM of bovine eyes treated with steroid (unpublished data). On the basis of this evidence, we tested whether PLAT upregulation can prevent and/or reverse GC-induced outflow resistance changes.

We have recently established a mouse model of steroid-induced change in outflow facility in which subconjuntival injection of triamcinolone acetonide (TA) decreases outflow facility after one week of injection [19]. In the present study we used adenoviral vector carrying sheep PLAT cDNA to upregulate PLAT expression as adenoviruses have been shown to transfect TM cells very efficiently [26,27].

We used the sheep PLAT gene as the induced transgene. Sheep tPA shares a 78% similarity with the mouse tPA and similarly the PLAT genes show a 78% similarity at the cDNA level. This difference in combination with the primers that were designed specifically for the sheep PLAT allows us to measure PLAT transgene transcription after RNA extraction of mouse angle tissues.

In addition we constructed our expression cassette to include a red fluorescent protein under IRES control. This has enabled us to visually observe whether the transgene was expressed in the TM. Despite the high concentration of adenovirus used in this experiment we were surprised to find that a number of eyes did not show any evidence of mCherry expression in the anterior chamber (AC). In addition in eyes that showed mCherry expression in the TM no other tissues showed similar expression in the AC. This is in contrast to other adenoviruses (with different transgenes) we have tested (data not shown) that often show extensive expression in the corneal endothelium and may indicate quick silencing of the transgene production in these tissues. Nonetheless to ensure that mCherry expression reflects PLAT expression we performed qPCR in tissues from both eyes that had significant as well as eyes with negligible mCherry expression. qPCR results confirmed that PLAT expression is undetectable in eyes without mCherry expression. Based on this finding, we analyzed eyes injected with AdPLAT as separate groups depending on their TM mCherry expression.

We initially asked whether adenovirus induced-PLAT overexpression can prevent outflow facility changes in mice. To address this question we administered AdPLAT concurrently (immediately after) subconjunctival TA injection. Adenoviral transgene expression occurs within 48-72 hours [27] (unpublished data) and thus precedes the changes in outflow facility that occur in mice after approximately one week of TA treatment. Determination of outflow facility at one week after TA and AdPLAT administration revealed that sheep PLAT overexpression in the mouse TM leads to a 60% increase in outflow facility. As expected this increase in outflow facility did not cause a significant reduction in IOP [19]. Similarly, administration of AdPLAT one week after TA injection increased outflow facility one week later, by 63%. This finding confirms that PLAT overexpression is not only sufficient to prevent outflow facility changes induced by steroids but can just also reverse the changes in this animal model.

In an effort to understand how tPA causes these changes in outflow facility we examined the expression levels of some of the genes that regulate the tPA pathway, as well as known effectors of tPA actions. As the amount of tissue that can be collected from a single mouse eye is rather limited for multiple PCR reactions we had to pool four eyes per group of genes analyzed. This could have resulted in a slight decrease of the variability between individual eyes. Despite this we were able to determine that PAI-1 expression was upregulated in eyes overexpressing sheep PLAT. Since tissue dissection inevitably contained some ciliary body in all eyes it is unclear whether PAI-1 was actually upregulated in the TM or in the ciliary processes (CPs). It is known that PAI-1 is produced in the CPs and secreted in the aqueous humor [28]. However, PAI-1 mRNA has also been detected in cultured human TM cells [29]. Prior reports have shown that PAI-1 levels are increased in patients with chronic open angle glaucoma [30], making the results of the current study potentially relevant to a wider number of glaucomas in addition to steroid-induced IOP elevation. In the present study we believe that PAI-1 upregulation is part of a feedback loop that attempts to control excessive tPA activation. PLAT overexpression seems to also upregulate expression of a number of pro-MMPs. We specifically show that it results in an eight-fold upregulation of MMP-2 and additional upregulation of MMP-9 and -13 which now become detectable.

MMPs are a family of zinc- and calcium-dependent enzymes able to degrade ECM components. In particular, two members of the MMP family, MMP-2 (gelatinase A) and MMP-9 (gelatinase B), are able to degrade major components of ECM like collagens type IV, V, VII and X, laminins, and fibronectin. Previous studies have shown that MMP-2 and MMP-9 may play major roles in the pathogenesis of steroid induced glaucoma as their levels were decreased in human TM cells treated with steroid [31]. In a perfused human organ culture injection of MMP-2 and MMP-9 led to a significantly increased outflow facility [5]. Decreased extracellular activities of MMPs were also reported in steroid treated human TM organ and cell cultures [16]. Altered levels of MMPs have also been described in the chamber angle and aqueous humor of patients with primary open angle glaucoma and exfoliation glaucoma [32,33]. Together these findings suggest the role of MMPs in the pathophysiology of steroid induced glaucoma as well as in other forms of glaucoma. In the present study, we found that overexpression of PLAT upregulated expression of MMP-2, -9 and -13 significantly compared to the levels found in TA only treated eyes. Similarly, tPA induced MMP-2 and MMP-9 upregulation has been reported in human cerebral microvascular endothelial cell culture [34] while increased activity and expression of MMP-9 by recombinant tPA have also been described in stroke [35,36].

MMP-13 is an interstitial collagenase that degrades collagen type I, collagen type III and collagen type IV. Collagen types I and II are the interstitial types that form banded fibrils of the trabecular core, the basement membrane of the trabecular beams and loose aggregates in the juxtacanalicular tissue [37]. Type IV collagen is another interstitial type of collagen, which has been shown to form a fine network around striated fibrils in the trabecular cores and linkage strands to the basement membranes [38]. Dexamethasone has been shown to upregulate collagen type I protein in the organ-cultured trabecular meshwork [39]. Although the association between MMP-13 and glaucoma is not strongly established, we investigated MMP-13 expression because PLAT gene disruption is known to affect MMP-13 expression in the liver of animals subjected to CCl(4) injury [40]. tPA induced MMP-13 upregulation in our study suggests that this MMP may also be involved in degrading ECM collagen in the TM.

Although upregulation of expression of pro-MMPs does not prove that these enzymes become active as a result of activation of tPA in the TM, it makes such a mechanism of action very likely. tPA has however been shown to work through other non-MMP related pathways in modulating ECM. For example, tPA mediated ECM modulation can be through plasmin which can directly degrade ECM components like laminin, fibronectin and elastin [41,42]. Determination of whether plasmin directly degrades ECM in the TM in this animal model will require additional work.

In summary, we have shown that overexpression of PLAT in TM of mouse eyes can both prevent and reverse the decrease in outflow facility caused by steroid treatment. The PLAT mediated increase in outflow facility is associated with upregulation of MMPs and occurs despite a compensatory increase in PAI-1 expression. These findings could have important therapeutic implications in the management for steroid induced glaucoma.
